# Chemical fertilizer reduction combined with organic fertilizer affects the soil microbial community and diversity and yield of cotton

**DOI:** 10.3389/fmicb.2023.1295722

**Published:** 2023-11-20

**Authors:** YingWu Shi, XinXiang Niu, BaoZhu Chen, ShengHai Pu, HongHong Ma, Pan Li, GuangPing Feng, XingWang Ma

**Affiliations:** ^1^Institute of Microbiology, Xinjiang Academy of Agricultural Sciences, Urumqi, Xinjiang, China; ^2^Key Laboratory of Agricultural Environment in Northwest Oasis of Ministry of Agriculture and Countryside, Urumqi, Xinjiang, China; ^3^Xinjiang Laboratory of Special Environmental Microbiology, Urumqi, Xinjiang, China; ^4^Institute of Soil, Fertilizer and Agricultural Water Conservation, Xinjiang Academy of Agricultural Sciences, Urumqi, Xinjiang, China

**Keywords:** cotton, Illumina MiSeq, organic fertilizer, reduced chemical fertilizer, soil microbial community, yield

## Abstract

**Introduction:**

The soil microbial community plays an important role in modulating cotton soil fertility. However, the effects of chemical fertilizer combined with organic fertilizer on soil chemical properties, microbial community structure, and crop yield and quality in arid areas are still unclear. This study aimed to explore the effects of different organic fertilizers on soil microbial community structure and diversity and cotton growth and yield.

**Methods:**

High-throughput sequencing was used to study the soil bacteria and fungi in different growth stages of cotton. The field fertilization experiment had five treatments.

**Results:**

The results indicated that the treatments of chemical fertilizer reduction combined with organic fertilizer significantly increased soil available nitrogen and phosphorus in cotton field. There were significant differences in the abundance of the bacterial and fungal communities in the dominant phyla among the treatments. At the phyla level, there were not significantly different in the diversity of bacteria and fungi among treatments. There were significant differences in the composition and diversity of bacterial and fungal communities during the entire cotton growth period (*p* = 0.001). The rhizosphere bacterial and fungal community structure was significantly affected by soil TK, NH_4_^+^, AK, TP, AN, and NO_3_^−^. The different fertilization treatments strongly influenced the modular structure of the soil bacterial and fungal community co-occurrence network. A reduction in chemical fertilizer combined with organic fertilizer significantly improved cotton stem diameter and seed yield, and the effect of the biological organic fertilizer on plant growth and yield formation was greater than that of ordinary organic fertilizer.

**Discussion:**

This study provide a scientific and technical basis for the establishment of environmentally friendly green fertilization technology for cotton in arid areas and the promotion of sustainable development of cotton industry.

## 1 Introduction

Xinjiang is the largest commodity cotton base in China (Niu et al., 2021). Applying chemical fertilizer increases the number of bolls per plant, reduces the bud and boll abscission rate, and increases cotton yields (Zheng et al., 2015). However, the long-term use of chemical fertilizers can lead to a deterioration in soil physicochemical properties, resulting in agricultural non-point source pollution, which can lead to sustained increases in fertilizer use and water, air, and soil pollution (Zhou et al., 2011). Excessive nitrogen fertilizer inputs not only waste resources, but also lead to environmental pollution and soil degradation (Chen J. S. et al., 2021).

It has become an urgent challenge in agricultural production to identify reasonable fertilization measures that reduce the harm caused by excessive soil fertilizer applications. Chemical fertilizer reduction combined with organic fertilizer is an environmental protection fertilization technology (Ye et al., 2020). Appropriate organic fertilizer substitution promotes crop yield, improves soil nutrients and protects soil ecology (Ding et al., 2021; Li et al., 2021a,b; Xiao et al., 2022).

Organic fertilizer combined with chemical fertilizer can reduce nutrient losses in cotton fields (Adeli et al., 2008), improve the fertilizer utilization rate, and increase soil organic matter content (Mensik et al., 2018; Wang et al., 2022). This fertilization method activate soil nutrients, improve soil microbial community structure and diversity (Guo et al., 2019; Zhang et al., 2019; Dong et al., 2020), and change rhizosphere soil enzyme activity. It enhance soil microbial activity, improve soil fertility, and reduce soil salinization and soil-borne diseases (Halihashi et al., 2020; Zhang et al., 2021). Then it improve crop root activity (Wang et al., 2022), antioxidant enzyme activity (Tang et al., 2018), and leaf pigment content (Wang N. et al., 2020; Wang Y. et al., 2020; Wang Q. Q. et al., 2020). It also increases water and fertilizer conservation and ultimately improves crop yield and stress resistance.

One study reported that 80% conventional fertilization combined with moderate amino acid, fulvic acid, and biogas slurry inputs produced the highest cotton yield and benefit (Li et al., 2019), and was significantly higher than that produced by conventional fertilization (Li et al., 2019). Previous studies have shown that when the amount of organic fertilizer was the same, and there was no significant difference in cotton yield between different types of organic fertilizer treatments (Tao et al., 2017; Li et al., 2019; Sun et al., 2020). The application of 70% chemical fertilizer combined with green manure did not reduce the nutrient accumulation, seed cotton yield and fertilizer utilization rate of cotton plant (Li et al., 2019). Furthermore, the agronomic efficiency of chemical fertilizer was significantly higher than that for the 100 and 85% chemical fertilizer treatments, and the seed cotton yield increased by nearly 30% (Sun et al., 2015; Li et al., 2021a,b). The improvement in phosphorus use efficiency after applying biological organic fertilizer is better than that achieved by applying ordinary organic fertilizer. The replacement of 40% chemical fertilizer with bio-organic fertilizer significantly increased cashmere length and yield (Sun et al., 2020). Chemical fertilizer combined with organic fertilizer also significantly increased the number of bacteria and actinomycetes, decreased the number of fungi, and increased urease, catalase, sucrose, and alkaline phosphatase activities (Wang N. et al., 2020; Wang Y. et al., 2020; Wang Q. Q. et al., 2020). Reducing nitrogen fertilization by 30% can reduce soil electrical conductivity and improve soil nutrient content, which will ultimately increase cotton yield and quality (Zhu et al., 2020). It can be seen that chemical fertilizer reduction combined with organic fertilizer is economically feasible. Few studies have investigated the effect of replacing chemical fertilizer with organic fertilizer on microorganisms and cotton growth traits and yield.

In this study, ordinary organic fertilizer, humic acid urea, biological bacterial fertilizer substitution, and conventional fertilization were taken as the research objects. Field experiments were used to investigate the effects of different fertilization regimes on cotton yield, soil nutrients, and the microbial community. In addition, the scientific and economic efficiency of combining chemical fertilizer reduction and organic fertilizer applications was also evaluated. The underlying hypotheses are that (I) the reduction of chemical fertilizer combined with organic fertilizer increases the content of soil nutrient in cotton fields; and (II) affects the community structures of soil microorganism over the whole cotton growth period.

## 2 Materials and methods

### 2.1 Field site and experimental design

The experiment was conducted at Xinjiang Academy of Agricultural Sciences in Korla City, Xinjiang, in 2020. The experimental area was located on Baotouhu Farm, Xinjiang Academy of Agricultural Sciences, Korla City, southern Xinjiang (E 85°52′, N 41°41′). The experimental area has a typical arid climate, the average annual rainfall is 56.20 mm. The tested soil was a medium fertility sandy loam soil, the soil organic matter in the 0–30 cm of soil above the plow layer was 10.24 g/kg, available nitrogen was 48.78 mg/kg, available phosphorus was 20.36 mg/kg, available potassium was 139.00 mg/kg, and the pH was 8.20.

The experiment consisted of five treatments (Supplementary Table S1): T1: no fertilizer application; T2: conventional fertilization (*CF*; NPK dosage: 714 kg/ha: 357 kg N/ha, 207 kg P/ha, and 150 kg K/ha); T3: 60% *CF* + 12,000 kg/ha organic fertilizer; T4: 46% *CF* + 428.4 kg/ha humic acid urea; and T5: 73% *CF* + 225 kg/ha bio-organic fertilizer. Each treatment was replicated three times and the area of each experimental plot was 40.5 m^2^. Organic fertilizer refers to sheep manure. The nutrient contents of sheep manure were N 0.192%, P2O5 1.16%, K2O 0.82%, humic acid 19.04%, organic matter 56.05%, water content 51.50%. Humic acid urea is a urea containing 0.12% humic acid. The nutrient contents of *Bacillus velezensis* were N 0.357%, P2O5 0.09%, K2O 0.04%, humic acid 2.00%, organic matter 1.94%. The bio-organic fertilizer is a bacterial fertilizer produced by fermentation of *Bacillus velezensis* BHZ-29, which we isolated from cotton fields (Zhang et al., 2018).

The cotton variety was Xinluzhong 66. The common organic fertilizer was used as a base fertilizer and applied to the 0–20 cm soil layer before sowing. The chemical fertilizers, humic acid urea, and bio-organic fertilizer were drip applied with water six times throughout the cotton growing season according to their fertilizer requirements (Niu et al., 2021).

### 2.2 Soil sampling and analysis of physicochemical properties

Soil samples from the 0–20 cm soil layer were collected by soil drilling at the cotton seedling stage (B), bud stage (M), flowering stage (H), boll opening stage (T) in 2020. There were three replicates per treatment, a five-spot sampling method for each treatment. Each replicate collected 500 g soil samples. Plant residues and stones were removed from the soil samples using a 2 mm mesh sieve. Some of the samples were placed in a 4°C refrigerator for the determination of soil microbial diversity, whereas the others were stored under natural air drying to determine the soil physicochemical properties. The SPAD (Soil and Plant Analyzer Development) value of cotton leaves was measured at bud stage, flowering stage and boll stage (Chen et al., 2011). The agronomic traits, yield, and yield related factors were measured at the cotton boll opening stage and the harvest stage. Ten cotton plants were randomly selected from each plot before boll opening. The number of fruit branches per plant, plant height, and boll number per plant were measured. The yield was measured after drying and the seed cotton yield for the plot was calculated. The lint yield and lint percentage were calculated after rolling and the cotton quality index was determined by the Cotton Quality Supervision and Testing Center of the Ministry of Agriculture and Rural Areas (Wan et al., 2018; Chen et al., 2020).

Soil moisture content (WCR) was determined by the drying method. Soil organic matter (OM) was determined using potassium dichromate dilution heat method, total nitrogen (TN) was quantified by a Kjeldahl nitrogen analyzer (multi N/C 2100 S, Analytik Jena, Jena, Germany), alkaline nitrogen (AN) was measured using the diffusion method, soil ammonium nitrogen (NH4+) and nitrate (NO3-) was determined by flow analyzer, total phosphorus (TP) was determined by the acid-soluble-molybdenum-antimony colorimetric method, effective phosphorus (AP) was determined using sodium bicarbonate extraction-molybdenum antimony antispectrophotometry, total potassium (TK) was measured using an NaOH alkali fusion-flame photometer, and available potassium (AK) was determined using the ammonium acetate extraction-flame photometric method (Ma et al., 2022). Each analysis was repeated three times.

### 2.3 Extraction and sequencing of soil microbial DNA

The soil microbial DNA was extracted according to the Power Soil DNA Isolation Kit instructions (MoBio Laboratories, Carlsbad, CA, United States). The extracted DNA was detected by 1% agarose gel electrophoresis and spectrophotometry, and the qualified samples were stored at −20°C until needed. Primers 338F (5’-ACTCCTACGGGAGGCAGCAG-3′) and 806R (5’-GGACTACHVGGGTWTCTAAT-3′) were used to amplify the V3–V4 region of bacterial 16S rRNA gene (Jones et al., 2021) and the primers for ITS gene sequencing of the fungi were ITS1F (5’-CTTGGTCATTTAGAGAAGTAA-3′) and ITS2 (5’-TGCGTTCTTCATCGATGC-3′) (Li S. et al., 2020; Li Y. et al., 2020). The above primers with barcode sequences were synthesized for the PCR amplification procedure. The PCR products were detected by 1.5% agarose gel electrophoresis, purified using a TIANgel nucleic acid purification kit (Tiangen, Beijing, China) and then used to construct a microbial diversity sequencing library. Paired-end sequencing was performed using the Illumina MiSeq high-throughput sequencing platform by Shanghai Majorbio Bio-pharm Technology Co., Ltd. (Shanghai, China).

### 2.4 Analysis method for the sequencing data

To improve the accuracy and reliability of the information analysis, the offline data were split into samples according to their Barcode sequence by QIIME1 (v 1.8.0) software. The data were filtered and spliced by Pear (v 0.9.6) software and the chimera sequences are removed using the Uchime method according to the Gold Database. Finally, the Vsearch (v 2.7.1) software UPARSE algorithm was used to cluster the high-quality sequences based on 97% consistency and the Silva128 database was used to annotate the OTUs (Operational Taxonomic Units) using the RDP Classifier algorithm. Then, the community composition of each sample was analyzed at the phylum, class, order, family, genus, and species levels to obtain the species composition and relative abundance at each taxonomic level (Shi et al., 2022). The α-diversity of each sample was calculated using Mothur 1.45.3 (Barouillet et al., 2022) and Excel 2007 (Microsoft, Redmond, WA, United States) software and included the Chao1 value, the Shannon index, and the Simpson index. Rarefaction curves were obtained from the ratio of the number of OTUs in the sample to the effective reads.

### 2.5 Data processing

Microsoft Excel 2007 was used for data pre-processing and presentation, and SPSS 20.0 (IBM Corp, Armonk, NY, United States) software was used for the one-way analysis of variance (ANOVA) and the Duncan method (α = 0.05) for multiple comparisons and difference testing. The data are shown as the mean ± standard error. A principal coordinate analysis (PCoA) and a permutation multi-factor analysis of variance (Adonis) based on the sample Bray-Curtis distance (OTU level) were performed by R Studio (version 4.0.3), and a dominant OTU heat map and a correlation heat map were constructed. Canoco 5.1 was used to draw a redundancy analysis diagram (redundancy analysis, RDA) (Dalu et al., 2021). Finally, the Spearman’s correlation coefficient was used to calculate the correlations among OTUs (relative abundance ≥0.1%) and OTUs with correlation coefficients of *r* ≥ 0.6 and *p* < 0.01 were selected as data sources to construct the co-occurrence network for soil bacteria using Gephi (version 9.2) (Tang et al., 2018).

## 3 Results

### 3.1 Soil properties in response to chemical fertilizer reduction combined with organic fertilizer

It can be seen from Supplementary Table S2 that the treatments of chemical fertilizer reduction combined with organic fertilizer increased the contents of soil organic matter (OM), total nitrogen (TN), total phosphorus (TP), total potassium (TK), available nitrogen (AN), available phosphorus (AP) and available potassium (AK) in cotton field. Compared with T1, T3, T4 and T5 treatments significantly increased the content of soil available nitrogen and available phosphorus in cotton fields. The content of soil available nitrogen and available phosphorus in T3 treatment was the highest, reaching 32.33 mg / kg and 32.35 mg/kg. The trend of soil TN, TP, AN and AK in different fertilization treatments was T3 > T5 > T2 > T4 > T1. The content of SOM in each growth period of T3, T4 and T5 treatments showed an increasing trend. Except for T5, the total nitrogen of each treatment increased first and then decreased, and the total nitrogen of each treatment at flowering stage was the highest. Total phosphorus changed little in each period, and total potassium showed an increasing trend. Available nitrogen, phosphorus and potassium showed a downward trend.

### 3.2 Soil microbial alpha diversity

The 16S rRNA gene and ITS sequencing results showed that 2,616,391 and 3,812,536 original sequences were obtained from 60 samples. The samples contained 34,107–73,318 bacterial and 38,032–74,915 fungal sequences. The minimum numbers of sequences (34,107 and 38,032, respectively) were used as the sampling depth for the bacteria and fungi. The number of OTUs after flattening was 2,685–3,576 and 183–372 for bacteria and fungi, respectively (Table 1). There was no significant difference in the Chao1 indexes, Shannon indexes, and Simpson indexes for the rhizosphere bacterial and fungal communities among treatments (Table 2), indicating that chemical fertilizer reduction combined with organic fertilizer did not significantly change soil microbial α-diversity. The ACE, Chao and Shannon indexes of soil bacteria in the boll opening stage decreased significantly, and the ACE, Chao and Simpson indexes of soil fungi in the flowering and boll opening stages increased significantly.

**Table 1:** Statistics of sample sequence of soil bacteria and funge under different treatments.

Treatment	Bacterial		Fungal	
	Sequences	OTUs	Sequences	OTUs
T1	540,418	6,731	769,780	770
T2	530,104	6,503	776,576	738
T3	511,787	6,645	744,029	746
T4	516,572	6,487	758,039	709
T5	517,510	6,660	764,112	774
Total	2,616,391	7,802	3,812,536	1,180

T1: no fertilizer application (CK); T2: Conventional fertilization (CF; NPK dosage: 714 kg ha−1: 357 kg N ha−1, 207 kg P ha−1, and 150 kg K ha−1); T3: 60% CF + 12,000 kg ha organic fertilizer (CFO; NPK dosage: 428.4 kg ha−1: 214.2 kg N ha−1, 124.2 kg P ha−1, and 90 kg K ha−1); T4: 46% CF + 428.4 kg ha humic acid urea (CFO; NPK dosage: 714 kg ha−1: 357 kg N ha−1, 207 kg P ha−1, and 150 kg K ha−1); T5: 73% CF + 225 kg ha bio-organic fertilizer (CFB; NPK dosage: 617.55 kg ha−1: 260.55 kg N ha−1, 207 kg P ha−1, and 150 kg K ha−1).

**Table 2:** Soil microbial diversity index of different treatment and different growth stages.

Microbe	Sampling time	Treatment	Ace	Chao	Shannon	Simpson	Coverage
Bacteria	B	T1	4536.67 ± 386.62^a^	4348.68 ± 71.06^a^	6.60 ± 0.04^a^	0.0046 ± 0.0009^a^	0.9683 ± 0.0038^a^
		T2	4117.57 ± 89.06^a^	4158.72 ± 110.67^a^	6.57 ± 0.06^a^	0.0052 ± 0.0012^a^	0.9680 ± 0.0016^a^
		T3	4358.96 ± 410.72^a^	4179.94 ± 203.59^a^	6.63 ± 0.02^a^	0.0041 ± 0.0005^a^	0.9688 ± 0.0025^a^
		T4	4596.39 ± 378.84^a^	4218.28 ± 62.83^a^	6.58 ± 0.10^a^	0.0059 ± 0.0025^a^	0.9694 ± 0.0039^a^
		T5	4376.90 ± 190.53^a^	4201.28 ± 147.43^a^	6.58 ± 0.04^a^	0.0048 ± 0.0005^a^	0.9655 ± 0.0005^a^
	M	T1	4489.46 ± 202.27^a^	4491.07 ± 241.34^a^	6.60 ± 0.03^a^	0.0041 ± 0.0006^a^	0.9806 ± 0.0023^a^
		T2	4380.59 ± 183.77^a^	4338.51 ± 201.31^a^	6.56 ± 0.06^a^	0.0044 ± 0.0002^a^	0.9803 ± 0.0016^a^
		T3	4234.93 ± 54.27^a^	4227.17 ± 95.44^a^	6.51 ± 0.04^a^	0.0056 ± 0.0013^a^	0.9799 ± 0.0005^a^
		T4	4504.83 ± 288.02^a^	4326.57 ± 131.56^a^	6.52 ± 0.07^a^	0.0044 ± 0.0007^a^	0.9788 ± 0.007^a^
		T5	4540.26 ± 87.55^a^	4506.48 ± 43.22^a^	6.63 ± 0.05^a^	0.0041 ± 0.0006^a^	0.9787 ± 0.0008^a^
	H	T1	4736.61 ± 342.15^a^	4482.58 ± 140.08^a^	6.59 ± 0.04^a^	0.0041 ± 0.0001^a^	0.9749 ± 0.0011^a^
		T2	4483.86 ± 181.42^a^	4534.25 ± 192.78^a^	6.60 ± 0.02^a^	0.0042 ± 0.0004^a^	0.9743 ± 0.0029^a^
		T3	4466.28 ± 152.33^a^	4444.78 ± 168.34^a^	6.62 ± 0.05^a^	0.0037 ± 0.0002^a^	0.9758 ± 0.0031^a^
		T4	4299.29 ± 157.52^a^	4325.31 ± 167.40^a^	6.54 ± 0.06^a^	0.0048 ± 0.0006^a^	0.9763 ± 0.0014^a^
		T5	4594.59 ± 117.60^a^	4445.61 ± 278.72^a^	6.58 ± 0.07^a^	0.0039 ± 0.0003^a^	0.9746 ± 0.0019^a^
	T	T1	4224.49 ± 94.45^a^	4183.12 ± 92.73^a^	6.46 ± 0.07^a^	0.0057 ± 0.0013^a^	0.9774 ± 0.0008^a^
		T2	4369.93 ± 353.65^a^	4106.09 ± 36.31^a^	6.40 ± 0.02^a^	0.0058 ± 0.0008^a^	0.9786 ± 0.0019^a^
		T3	4170.23 ± 300.76^a^	4135.48 ± 279.01^a^	6.47 ± 0.07^a^	0.0056 ± 0.0005^a^	0.9754 ± 0.0014^a^
		T4	4285.48 ± 348.73^a^	4081.21 ± 240.30^a^	6.42 ± 0.06^a^	0.0057 ± 0.0008^a^	0.9771 ± 0.0012^a^
		T5	4124.10 ± 75.04^a^	4092.05 ± 135.40^a^	6.46 ± 0.03^a^	0.0051 ± 0.0003^a^	0.9789 ± 0.0020^a^
Fungi	B	T1	282.49 ± 27.84^a^	289.42 ± 19.71^a^	2.93 ± 0.37^a^	0.1320 ± 0.0467^a^	0.9993 ± 0.0002^a^
		T2	248.09 ± 2.23^a^	246.76 ± 4.61^a^	2.54 ± 0.22^a^	0.2085 ± 0.0249^a^	0.9994 ± 0.0001^a^
		T3	240.42 ± 13.30^a^	238.33 ± 8.38^a^	2.22 ± 0.49^a^	0.3180 ± 0.1499^a^	0.9991 ± 0.0002^a^
		T4	254.79 ± 37.75^a^	252.25 ± 36.41^a^	2.64 ± 0.39^a^	0.2158 ± 0.0887^a^	0.9994 ± 0.0001^a^
		T5	264.64 ± 46.87^a^	265.95 ± 50.00^a^	2.94 ± 0.09^a^	0.1298 ± 0.0155^a^	0.9994 ± 0.0002^a^
	M	T1	265.26 ± 31.31^a^	274.97 ± 34.69^a^	2.37 ± 0.19^a^	0.2754 ± 0.0731^a^	0.9994 ± 0.0002^a^
		T2	290.41 ± 37.62^a^	288.43 ± 39.52^a^	2.67 ± 0.16^a^	0.2232 ± 0.0620^a^	0.9993 ± 0.0003^a^
		T3	275.90 ± 46.58^a^	278.92 ± 50.87^a^	3.08 ± 0.37^a^	0.1331 ± 0.0414^a^	0.9994 ± 0.0003^a^
		T4	265.84 ± 28.97^a^	268.43 ± 27.74^a^	2.67 ± 0.12^a^	0.1787 ± 0.0169^a^	0.9994 ± 0.0002^a^
		T5	293.79 ± 16.28^a^	294.81 ± 16.68^a^	2.78 ± 0.19^a^	0.1807 ± 0.0217^a^	0.9993 ± 0.0001^a^
	H	T1	309.92 ± 25.09^a^	314.01 ± 23.06^a^	2.27 ± 0.15^a^	0.3362 ± 0.0416^a^	0.9993 ± 0.0001^a^
		T2	341.50 ± 71.90^a^	340.29 ± 75.35^a^	2.30 ± 0.79^a^	0.3279 ± 0.1976^a^	0.9992 ± 0.0003^a^
		T3	341.95 ± 5.16^a^	337.49 ± 3.27^a^	2.34 ± 0.30^a^	0.3209 ± 0.0807^a^	0.9993 ± 0.0001^a^
		T4	335.95 ± 23.39^a^	340.77 ± 25.87^a^	2.37 ± 0.25^a^	0.3122 ± 0.0796^a^	0.9993 ± 0.0001^a^
		T5	332.84 ± 15.08^a^	326.19 ± 7.73^a^	2.34 ± 0.13^a^	0.2885 ± 0.0301^a^	0.9992 ± 0.0001^a^
	T	T1	352.05 ± 36.79^a^	360.53 ± 30.73^a^	2.44 ± 0.20^a^	0.3012 ± 0.0423^a^	0.9992 ± 0.0002^a^
		T2	349.80 ± 26.72^a^	341.89 ± 28.35^a^	2.35 ± 0.46^a^	0.2709 ± 0.1266^a^	0.9992 ± 0.0001^a^
		T3	341.93 ± 23.17^a^	342.91 ± 27.11^a^	2.47 ± 0.52^a^	0.2547 ± 0.0914^a^	0.9992 ± 0.0001^a^
		T4	336.92 ± 27.30^a^	339.55 ± 34.77^a^	2.13 ± 0.14^a^	0.3555 ± 0.0366^a^	0.9991 ± 0.0002^a^
		T5	293.64 ± 55.99^a^	313.67 ± 63.39^a^	2.30 ± 0.01^a^	0.2917 ± 0.0349^a^	0.9993 ± 0.0002^a^

Values indicate mean ± SE (*n* = 3). Different superscript letters in the columns represent significant differences among fertilizer treatments according to one-way ANOVA (Duncan’s test, *p* < 0.05). The abbreviations T1, T2, T3, T4, and T5 are as defined in the footnote to Table 1. B: seedling stage; M: bud stage; H: flowering stage; T: boll opening stage.

### 3.3 Soil microbial community composition and structure

**Figure 1:**
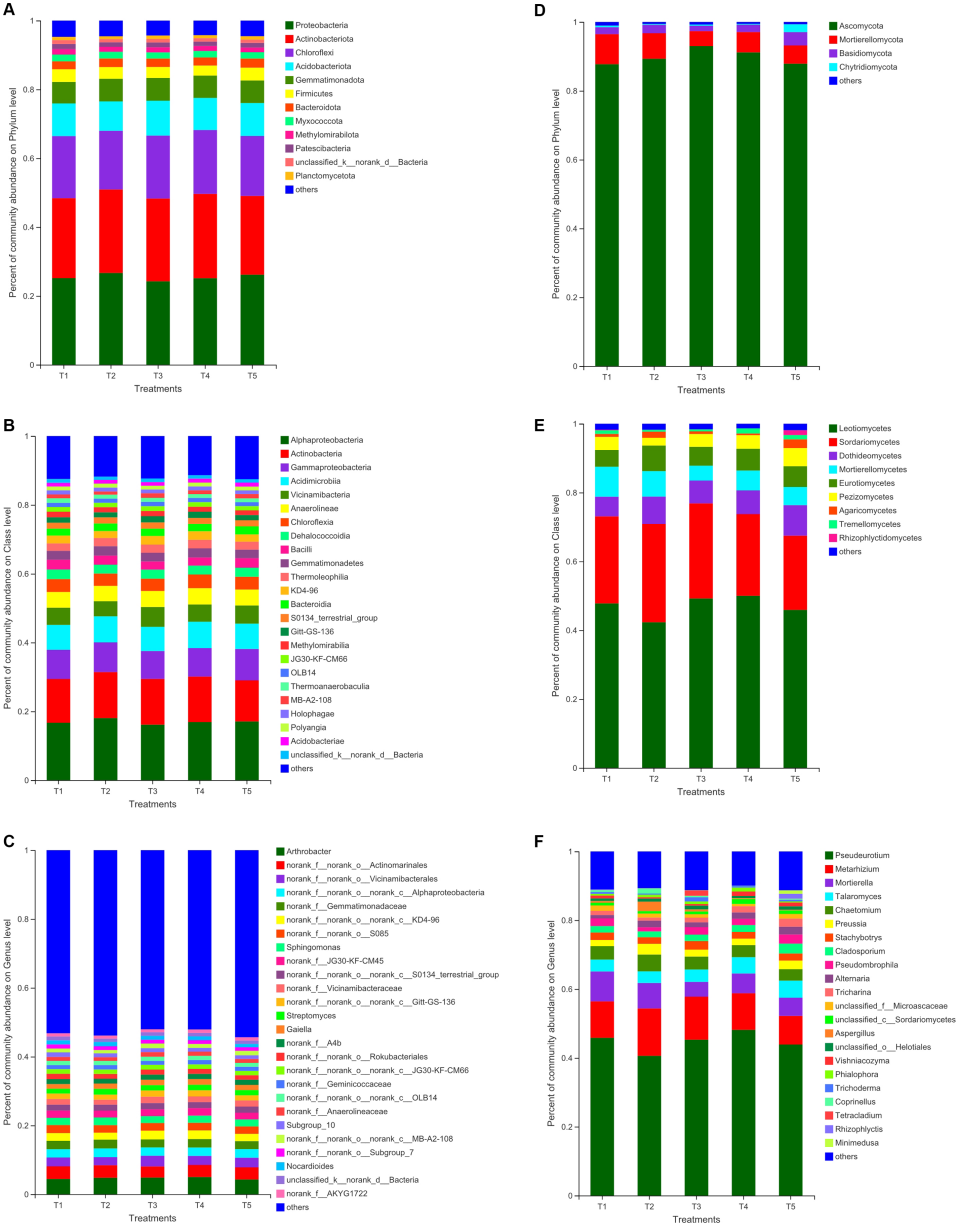
Relative abundances of bacterial **(A–C)** and fungal **(D–F)** taxa at the phylum, class, genus level, respectively. T1: no fertilizer application (CK); T2: Conventional fertilization (CF; NPK dosage: 714 kg ha-1: 357 kg N ha-1, 207 kg P ha-1, and 150 kg K ha-1); T3: 60% CF + 12,000 kg ha organic fertilizer (CFO; NPK dosage: 428.4 kg ha-1: 214.2 kg N ha-1, 124.2 kg P ha-1, and 90 kg K ha-1); T4: 46% CF + 428.4 kg ha humic acid urea (CFH; NPK dosage: 714 kg ha-1: 357 kg N ha-1, 207 kg P ha-1, and 150 kg K ha-1); T5: 73% CF + 225 kg ha bio-organic fertilizer (CFB; NPK dosage: 617.55 kg ha-1: 260.55 kg N ha-1, 207 kg P ha-1, and 150 kg K ha-1).

Among the five fertilization treatments, organic fertilizer combined with chemical fertilizer treatments (T3, T5) did not change the community structure of soil bacteria and fungi, but affected the bacterial abundance at different species classification levels (Figure 1). Among the five fertilization treatments, Proteobacteria, Actinobacteriota, Chloroflexi, Acidobacteriota, Gemmatimonadota, Firmicutes, Bacteroidota, and Ascomycota, Mortierellomycota, Basidiomycota, and Chytridiomycota were the dominant phyla in the bacterial and fungal communities, respectively (Figures 1A,D). Alphaproteobacteria, Actinobacteria, Gammaproteobacteria, Acidimicrobiia, Anaerolineae, Vicinamibacteria, Chloroflexia, Gemmatimonadetes, Bacilli, Dehalococcoidia, Thermoleophilia, Bacteroidia. KD4-96 was the dominant class of bacterial community (Figures 1B,E). The class with relative abundances greater than 2% in the T1, T2, T3, T4, and T5 treatments accounted for 73.11, 74.62, 73.11, 74.48, 73.76, 96.19, 95.91, 97.02, 96.69, and 92.94% of the total bacterial and fungal class, respectively (Figures 1B,E). With regards to the dominant genera of the bacterial and fungal community (relative abundance >1%), the T3 and T5 treatments increased the relative abundance of norank_f__norank_o__Vicinamibacterales, norank_f__Vicinamibacteraceae, norank_f__norank_o__norank_c__MB-A2-108 and Preussia compared to T1 and T2, but the norank_f__Gemmatimonadaceae and *Mortierella* relative abundances decreased (Figures 1C,F).

### 3.4 Dynamics of the soil microbial communities

**Figure 2:**
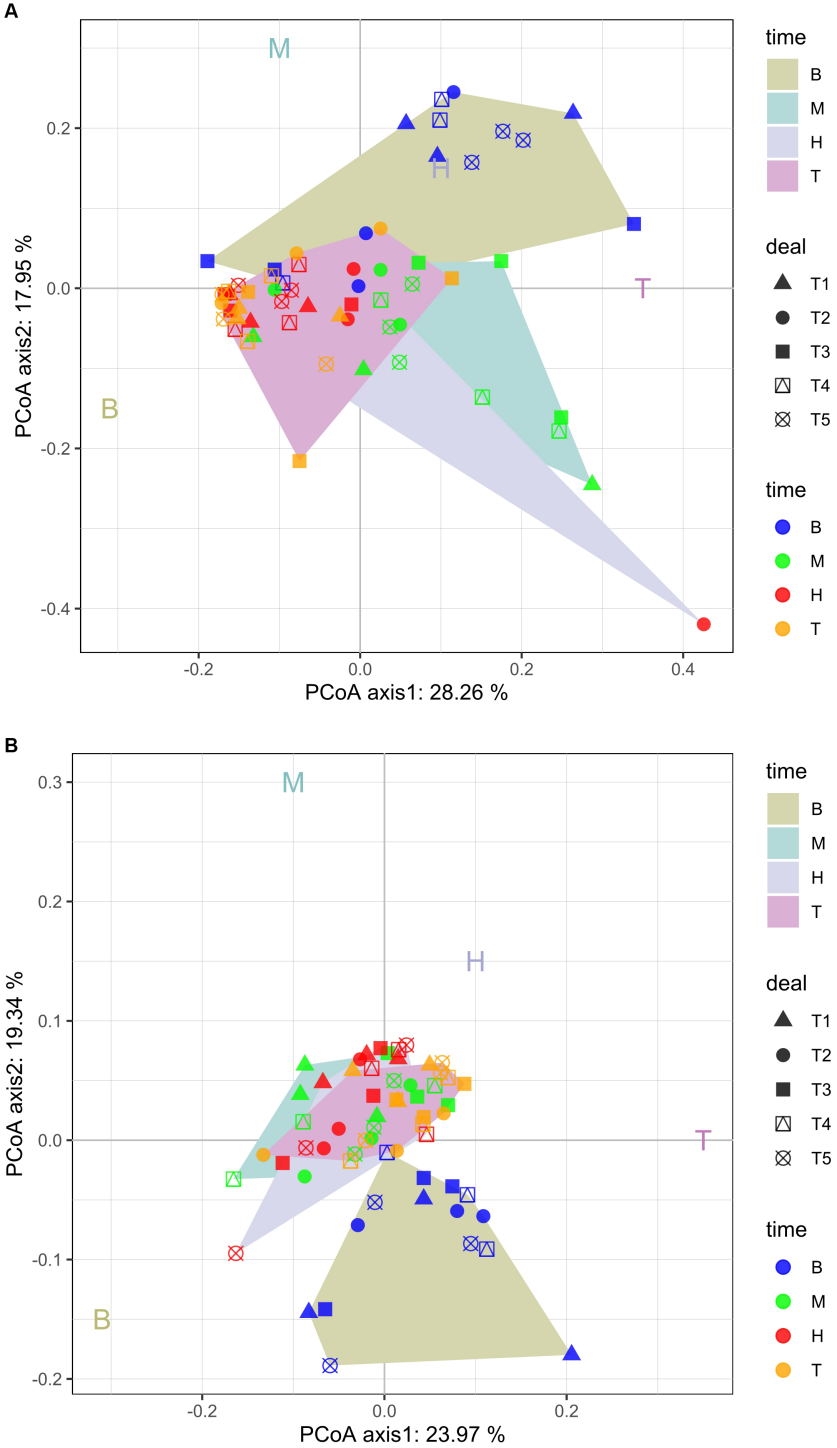
Principal coordinate analysis (PCoA) plots of bacterial **(A)** and fungal **(B)** community composition at the OTU level. T1, T2, T3, T4, and T5 are as defined in the Figure 1 legend. B: seedling stage: M: bud stage: H: flowering stage: T: boll opening stage.

The PCoA analysis showed that there are seasonal differences in the bacterial and fungal community structure among the samples (Figures 2A,B). The PC1 axis for bacteria and fungi explained 23.97 and 28.26% of the difference, respectively, and the PC2 axis explained 19.34 and 17.95%, respectively. Together, they explained 43.31 and 46.21% of the variability, respectively (Figures 2A,B). Figure 2 also showed that the dispersion degree of the samples at the bud, flowering, and boll stages was low. However, the dispersion degree was high for the samples at the seedling stage. There were significant differences in the community structures of the bacteria and fungi over the whole cotton growth period. The bacteria samples were highly clustered on the PC1 axis, which showed that the rhizosphere bacteria were highly similar in the samples, but the fungi were very different at the flowering stage.

The cluster analysis (Supplementary Figure S1) showed that there were significant differences in bacterial and fungal community composition among the samples at the bud, flowering, and boll stages, while the samples at the seedling stage were clustered on the same branch. The growth period had significant effects on the bacterial floras Actinobacteria, Gammaproteobacteria, Acidimicrobiia, Chloroflexia, Gemmatimonadetes, Dehalococcoidia, and Bacteroidia, and the fungal floras Leotiomycetes, Sordariomycetes, Mortierellomycetes, Eurotiomycetes, and Pezizomycetes were dominant in the five treatments (Supplementary Figure S2).

### 3.5 Correlation between bacterial and fungal abundance and soil properties

The Spearman’s correlation heatmap analysis indicated that the soil physicochemical factors affecting the soil bacterial and fungal communities were TK, NH4, AK, TP, and AN and TP, AN, NO3, and TK, respectively (Supplementary Figure S3). The effect of soil physicochemical factors on *Bacillus* was weak and only TK, NH4, TP, and AN were significant factors. *Pseudounas* was sensitive to most soil physicochemical factors, except for NH4+ and NO3-. The physical and chemical factors OM and NH4+ had significant effects on *Microascus*, and WCR and NO3 had significant effects on *Fusarium*, and NO3-had significant effects on *Tricharina* (Supplementary Figures S3A,B).

**Figure 3:**
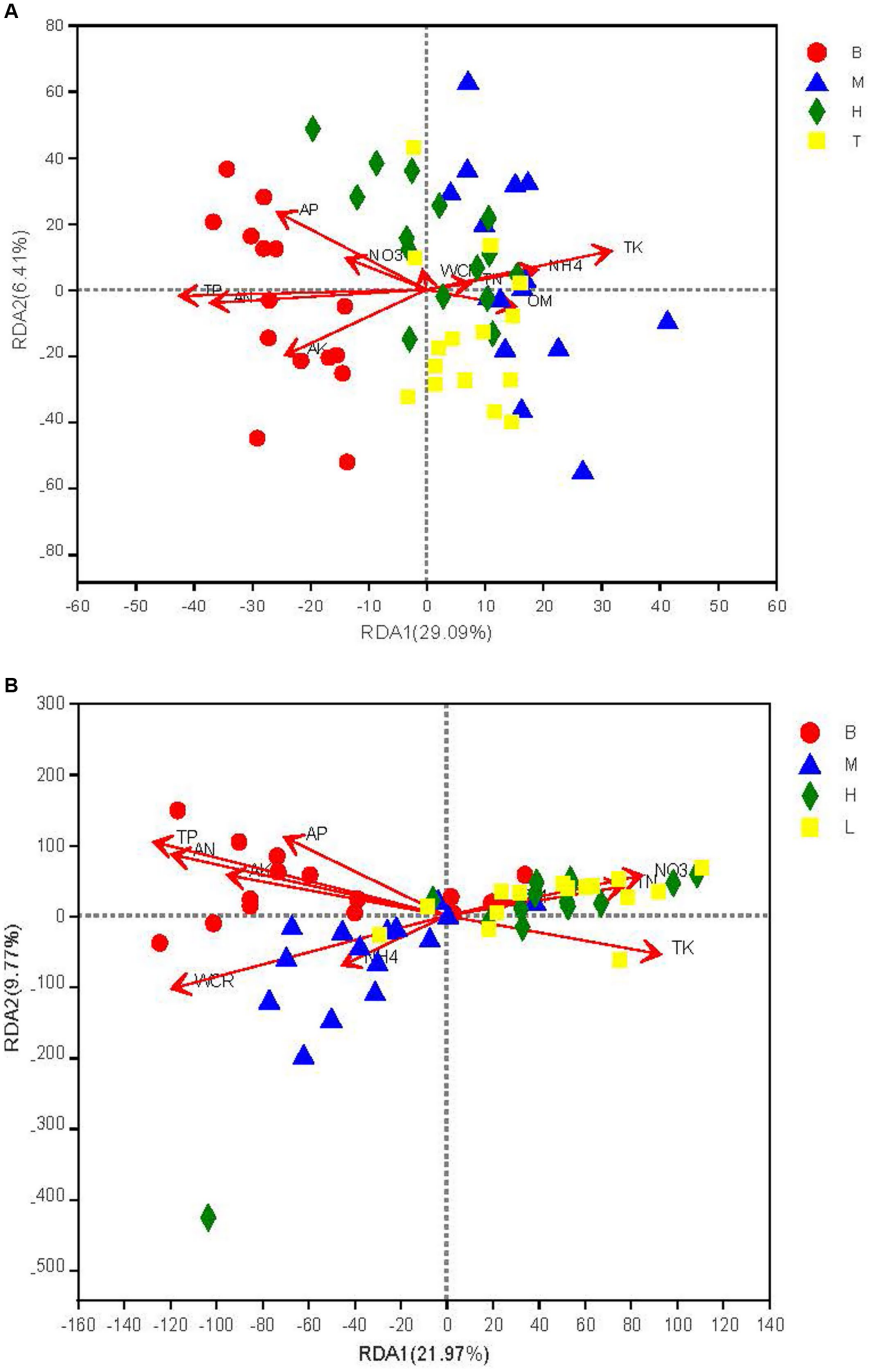
Redundancy analysis (RDA) of bacterial **(A)** and fungal **(B)** communities with soil chemical properties. WCR, electrical conductivity; OM, soil organic matter, NO3, electrical conductivity, NH4, soil organic matter.TN. total nitrogen: TP. total phosphorus: TK, total potassium; AN, total nitrogen, AP. total phosphorus: AK, total potassium. T1, T2, T3, T4, and T5 are as defined in the Figure 1 legend. B: seedling stage; M: bud stage, H: flowering stage. T. boll opening stage.

The RDA analysis showed that soil physicochemical factors explained 35.50% of the differences in the community structure of the dominant bacteria and fungi, and that RDA1 and RDA2 explained 29.09, 21.97 and 6.41%, 9.77% of the bacterial and fungal differences, respectively (Figure 3). Total phosphate (*R*^2^ = 0.75, *p* = 0.0000), TK (*R*^2^ = 0.47, *p* = 0.0000), and AN (*R*^2^ = 0.59, *p* = 0.0000) for the bacterial community and TP (*R*^2^ = 0.17, *p* = 0.0012), TK (*R*^2^ = 0.10, *p* = 0.0139), and AN (R^2^ = 0.15, *p* = 0.0022) for the fungal community reached significant levels (Figures 3A,B).

### 3.6 Soil microbial network structure and composition analysis

**Figure 4:**
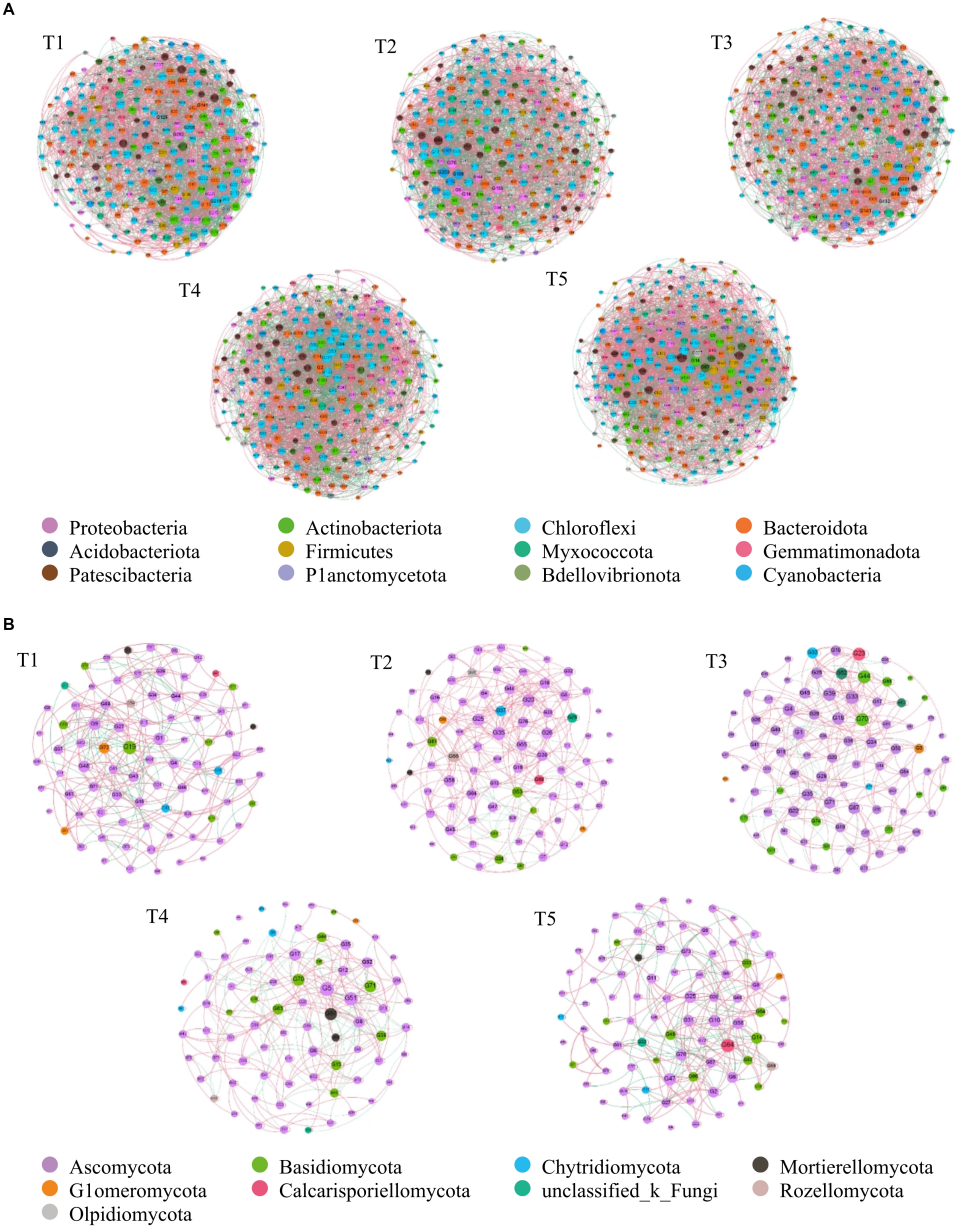
The co-occurrence network of soil bacterial and fungal community composition in a cotton field with different fertilization treatments on the phylum level. **(A)** bacterial community, **(B)** fungal community. Each node denotes a bacterial or a fungal OTU (defined at a 97% similarity level); each edge linking two nodes represents a positive (pink line) or negative (black line) relationship. OTUS are colored by different phylum. The size of each node is proportional to the number of connections. A connection between two nodes is a statistically significant (*p* < 0.01) and strong (*r* > 0.60) correlation. The percentage of positive links in every network: A 90.08%, B 73.87%.

The soil bacterial and fungal co-occurrence networks under the T1, T2, T3, T4, and T5 treatments were composed of 251, 253, 247, 254, 361, and 84, 85, 87, 79, 84 nodes, respectively, and 3,698, 3,403, 3,071, 3,776, and 4,291, 308, 337, 332, 305, 297 had highly significantly positively correlated edges, with average degrees of 28.337, 26.901, 24.866, 29.732, 32.881, and 7.333, 7.929, 7.632, 7.722, 7.071, respectively (Figures 4A,B). The modular indexes were 3.249, 5.458, 2.793, 3.498, 2.955, and 1.798, 0.638, 0.757, 0.316, 1.965, respectively. A modularity index >0.4 indicates that the co-occurrence network has a modular structure.

Figure 4A also indicated that the bacterial co-occurrence networks for T1, T2, and T4 were similar, whereas the bacterial co-occurrence networks for T5 were relatively complex. The fungal co-occurrence networks for T1, T4, and T5 were similar, as were the fungal co-occurrence networks for T2 and T3 (Figure 4B).

Supplementary Table S3 showed that the proportions of the bacterial and fungal groups in the co-occurrence network were different in each treatment. The Proteobacteria and Actinobacteriota proportions were the largest in the bacterial co-occurrence network and the Ascomycota and Basidiomycota proportions were largest in the fungal co-occurrence network. The bacterial groups in each treatment were basically the same, but the number of fungal groups was lower in T3. In addition. Proteobacteria, Actinobacteriota, Chloroflexi, Bacteroidota, Acidobacteriota, Firmicutes, and Myxococcota were the dominant genera in the soil bacterial co-occurrence network and Pseudeurotium, Metarhizium, Talaromyces, Trichoderma, Preussia, Chaetomium, and Mortierella were the dominant genera in the soil fungal co-occurrence network.

### 3.7 Cotton agronomic traits, quality and yield

The T5 treatment produced the highest yield (Table 3). The four fertilization treatments significantly increased the boll number per plant and the seed cotton yield compared to T1 (Table 3). Compared to T2, the stem diameter and seed cotton yield significantly increased under the chemical fertilizer reduction combined with organic fertilizer treatments, but there was no significant difference in plant height, leaf number, SPAD, single boll weight, and lint percentage. This showed that organic fertilizer promoted cotton plant growth and boll formation, but had no significant effect on cotton yield per plant. The raw cotton yields of the chemical fertilizer reduction combined with organic fertilizer treatments were in the order T5 > T4 > T3 and the differences among the three treatments were significant. The raw cotton yields of T4 and T5 were 5.27 and 12.10% higher than those of T2, respectively, when T2 was reduced by 50 and 54%, combined with 428.4 kg hm^−2^ humic acid urea (T4) and 225 L hm-2 *Bacillus velezensis* fertilizer (T5), respectively, and there was no significant effect on cotton quality.

**Table 3:** Effects of different fertilizer treatments on agronomic characters, quality and yield of cotton.

Parameters	Treatments				
	T1	T2	T3	T4	T5
Plant height	63.57 ± 12.28^a^	60.93 ± 8.04^a^	66.00 ± 9.46^a^	67.07 ± 10.97^a^	66.40 ± 6.49^a^
Number of blades	12.87 ± 1.46^b^	13.07 ± 1.80^a^	14.00 ± 1.65^a^	14.07 ± 1.28^a^	13.93 ± 1.91^a^
Stem diameter	9.14 ± 0.88^a^	8.91 ± 1.11^b^	9.65 ± 1.33a	9.71 ± 1.15^a^	9.87 ± 1.51^a^
SPAD	51.0 ± 9.64a	54.39 ± 12.09^a^	49.81 ± 11.95^a^	50.95 ± 14.44^a^	53.78 ± 14.22^a^
Boll number per plant	5.55 ± 1.35^b^	9.89 ± 0.70^a^	9.33 ± 0.67^a^	10.45 ± 3.24^a^	10.11 ± 2.37^a^
Boll weight	5.42 ± 0.27^a^	5.77 ± 0.20^a^	5.68 ± 0.11^a^	5.80 ± 0.18^a^	5.70 ± 0.35^a^
Lint percent	44.64 ± 0.38^a^	44.39 ± 0.54^a^	44.11 ± 0.16^a^	44.52 ± 0.43^a^	44.12 ± 0.53^a^
Raw cotton yield	309.47 ± 3.30^d^	427.68 ± 8.83^c^	418.43 ± 5.44^c^	450.2 ± .83^b^	479.43 ± 9.48^a^

Values indicate mean ± SE (*n* = 3). Different superscript letters in the columns represent significant differences among fertilizer treatments according to one-way ANOVA (Duncan’s test, *p* < 0.05). The abbreviations T1, T2, T3, T4, and T5 are as defined in the footnote to Table 1.

## 4 Discussion

### 4.1 Effects of fertilization on soil physicochemical properties

The proportion of soil available nutrients compared to the total soil nutrients is one of the important indicators used to evaluate the effectiveness of soil nutrients (Zhao and Huang, 2022). Reasonable combinations of organic and inorganic fertilizers can improve the soil physicochemical properties. In this study, we have demonstrated that reductions in chemical fertilizer combined with organic fertilizer could increase soil nutrients, such as AP, SOM and TN, which was consistent with previous studies on agricultural soils (Lu et al., 2009; Hossain et al., 2021; Yang W. N. et al., 2022). The increase in AP content by the fertilizer combined with organic fertilizer suggests that the organic fertilizer provided a large amount of soil organic matter (SOM), and then, organic matter decomposes under the action of microorganisms to produce a large number of organic acids, which can activate soil phosphorus, thereby increasing the soil AP content (Alori et al., 2017).

### 4.2 Effects of fertilization on the diversity and composition of soil microbial community

In microbial diversity studies, the greater the Shannon index, the higher the microbial community evenness; the higher the Chao1 and Ace indices, the higher the richness of the microbial communities (Hartmann et al., 2006). We also found that chemical fertilizer reduction combined with organic fertilizer increased soil bacterial richness and diversity, and reduced fungal richness and diversity (Table 2), which was consistent with previous studies that showed increased richness and diversity of the bacterial community, and decreased richness and diversity of the fungal community (Liu et al., 2021; Jin et al., 2022).

Microorganisms are an important part of the soil ecosystem. Their community composition and diversity show certain dynamic changes at different growth stages and under different nutritional statuses, fertilization measures, and tillage modes (Yang and Crowley, 2000; Wang et al., 2016; Liu et al., 2020; Iqbal et al., 2022). They are important indicators for evaluating soil fertility and are related to the occurrence of plant soil-borne diseases, continuous cropping obstacles, and soil acidification and salinization (Yao and Wu, 2010; Shen et al., 2016; Gao et al., 2021; Song et al., 2022). We found that there was little differentiation in the composition of bacterial or fungal communities among different fertilization treatments (Figures 2A,B). The reason might be that the soil organic matter content and microbial community diversity of long-term continuous cropping cotton field were low, and the organic matter content in fertilization treatment was not high.

### 4.3 Correlation between microorganism and soil physicochemical properties

Soil can directly provide the necessary environmental conditions for the survival of microorganisms (Santoyo et al., 2017). Organic fertilizer application can not only increase the total amount of soil nutrients such as soil total organic carbon, total nitrogen, and total phosphorus but can also increase the content of available soil nutrients (alkali nitrogen, available phosphorus, available potassium) (Liu et al., 2014; Qaswar et al., 2020). TP, TK, AN is significantly associated with the bacterial and fungal communities and is one of the main factors promoting changes in the soil microbial community (Dong et al., 2014; Hu et al., 2019; Chen Y. et al., 2021; Li et al., 2021a,b; Zhao et al., 2021). Proteobacteria and Ascomycota is a dominant taxon in the bacterial and fungal community, separately and is positively correlated with the soil nitrogen, phosphorus and potassium pool; therefore, TP, TK, AN has been proven to drive changes in the soil bacterial and fungal community (Landesman and Dighton, 2010; Zhong et al., 2010; Ducousso-Détrez et al., 2022). In the present study, TP, TK, and AN were found to be the key determinants driving changes in the bacterial and fungal community in this study (Figures 3A,B; Supplementary Figures S3A,B), which was consistent with previous studies on soils (Landesman and Dighton, 2010; Zhong et al., 2010; Ducousso-Détrez et al., 2022).

Another study reported that SOM were the major driver on structuring soil microbial communities across land uses, while soil bacterial communities were more sensitive to variations in SOM and geochemical characteristics compared with fungi (Bahadori et al., 2022). This is inconsistent with our results and suggests that the effect of chemical fertilizer reduction combined with organic fertilizer on microbial community structure is not the only factor. This makes it difficult to assess the full effect of their nutritional potential on the soil microbial community in a short-term study period (Zhang et al., 2021). Therefore, the short-term effect of chemical fertilizer reduction combined with organic fertilizer on soil microbial community may be smaller than chemical fertilizer.

### 4.4 Co-occurrence networks for soil microorganisms in cotton fields

The network complexity became gradually more complicated as soil nutrient levels increased (Liao et al., 2020; Wang et al., 2021; Ye et al., 2021), indicating that the increase in the co-occurrence network complexity in the T5 treatment was closely related to the increases in SOM, TN, TP, and AP contents (Supplementary Table S2). The proportion of Proteobacteria in the T5 co-occurrence network was higher than that for the other treatments, but the Patescibacteria, Chloroflexi, and Planctomycetota proportions were lower than in the other treatments in our study (Supplementary Table S3). Proteobacteria are mainly distributed in the upper humus layer of farmland soil or the rhizosphere and are enriched in environments with high levels of active organic carbon (Pinhassi and Berman, 2003; Sagova-Mareckova et al., 2016). In contrast, Chloroflexi are oligotrophic bacteria with slow growth characteristics (Feng et al., 2022) and are ubiquitous in nutrient-poor soils (Wang N. et al., 2020; Wang Y. et al., 2020; Wang Q. Q. et al., 2020). When the substrate concentration of the microbial environment increased, the nutrient-rich bacteria replaced the oligotrophic bacteria and colonize the nutrient-rich environment, which strongly suggested that the T5 treatment improved the growth of nutrient-rich bacteria and restricted the growth of oligotrophic bacteria (Wang N. et al., 2020; Wang Y. et al., 2020; Wang Q. Q. et al., 2020).

The dominant groups are considered to be important drivers of microbial community structure and function (Banerjee et al., 2018). Zhang et al. (2017) found that Acinetobacter and Pseudomonas were the core functional bacteria in acid red soil paddy fields and that their relative abundances increased by 5.7–10 times over 31 years, indicating that bacterial communities experienced ecological succession, and that dominant bacteria occupied specific niches and had specific functions. In this study, Proteobacteria, Actinobacteriota, Chloroflexi, and Pseudeurotium, and Metarhizium and Talaromyces were the core genera of soil bacteria and fungi in the co-occurrence networks.

### 4.5 Effects of fertilization on agronomic traits and cotton yield

Combined applications of chemical fertilizer and organic fertilizer can regulate the release and intensity of soil and fertilizer nutrients, which means that crops can obtain stable and balanced amounts of nutrients at all growth stages (Zhao et al., 2016; Muhammad et al., 2020). This study showed that the chemical fertilizer reduction combined with organic fertilizer treatments increased cotton yields by different degrees, which is consistent with previous studies on cotton field soils (Bai et al., 2014). Previous studies have shown that the ratio of chemical fertilizer to organic fertilizer is related to soil fertility and climatic conditions (Ye et al., 2020). When soil fertility is high, increasing the proportion of organic fertilizer can promote reproduction by microorganisms, improve soil structure, and increase crop yields (Ye et al., 2020). Bioorganic fertilizers can replace 23–52% of nitrogen fertilizers without causing a loss of yield (Rose et al., 2014).

In this study, the effect of fertilizer reduction combined with bacterial fertilizer on yield increase was clear, indicating that the nutrient release law for organic fertilizer was consistent with the nutrient demand law for cotton growth. At the same time, crop yield was significantly correlated with soil nutrients, microorganisms, and related enzyme activities (Jiang et al., 2017). Therefore, reductions in chemical fertilizer and reasonable applications of organic fertilizer could effectively control the number of soil microorganisms, improved soil enzyme activity and soil fertility. At present, soil nutrient imbalances in cotton fields caused by unreasonable fertilization is common in Xinjiang (Gong et al., 2012; Yang J. Y. et al., 2022). The fertilization structure can be adjusted and optimized to reduce chemical fertilizer application rates through the combined use of chemical fertilizer and organic fertilizer. In this study, reducing chemical fertilizer by 30% combined with 12,000 kg·hm^−2^ common organic fertilizer or 225 kg·hm^−2^ bio-organic fertilizer produced the highest yields, but the continuous application of organic fertilizer changed soil fertility. Therefore, future research should investigate the most appropriate proportion of organic fertilizer to apply to a field when combined with a chemical fertilizer application.

## 5 Conclusion

This study indicated that the reduction of chemical fertilizer combined with organic fertilizer significantly increased the content of soil available nitrogen and phosphorus in cotton fields, and that total and available nitrogen, phosphorus and potassium contents in the chemical fertilizer reduction combined with organic fertilizer treatments were basically stable in the cotton fields. The application of chemical fertilizer reduction combined with organic fertilizer significantly affected the community structures of the bacteria and fungi over the whole cotton growth period, without significantly changing the soil microbial alpha diversity. The different fertilization treatments strongly influenced the modular structure of the soil bacterial and fungal community co-occurrence network. A reduction in chemical fertilizer combined with organic fertilizer significantly improved cotton stem diameter and seed yield, and the effect of the biological organic fertilizer on plant growth and yield formation was greater than that of ordinary organic fertilizer.

## Data availability statement

The datasets presented in this study can be found in online repositories. The sequences from rhizosphere bacterial samples are available at NCBI SRA: BioProject PRJNA876230.

## Author contributions

YS: Formal analysis, Investigation, Writing – original draft. XN: Funding acquisition, Investigation, Resources, Writing – original draft, Writing – review & editing. BC: Investigation, Resources, Writing – original draft. SP: Investigation, Resources, Writing – review & editing. HM: Data curation, Formal analysis, Investigation, Writing – original draft. PL: Data curation, Resources, Writing – review & editing. GF: Formal analysis, Investigation, Writing – review & editing. XM: Formal analysis, Software, Supervision, Writing – review & editing.
