# Supplementary material for: Chemical fertilizer reduction combined with organic fertilizer affects the soil microbial community and diversity and yield of cotton
Source: Front Microbiol. 2023 Nov 20;14:1295722. doi: 10.3389/fmicb.2023.1295722 (PMC10694218; doi:10.3389/fmicb.2023.1295722)
Supplement: Supplementary file 1 [file Table_1.docx]

Table S1 The fertilizer rate in all treatments (kg ha^-1^)

| Treatment | N | P_2_O_5_ | K_2_O | Common organic fertilizer | Humic acid urea | Bio-organic fertilize |
| --- | --- | --- | --- | --- | --- | --- |
| T1 | 0.00 | 0.00 | 0.00 | 0.00 | 0.00 | 0.00 |
| T2 | 357.00 | 207.00 | 150.00 | 0.00 | 0.00 | 0.00 |
| T3 | 214.20 | 124.20 | 90.00 | 400.00 | 0.00 | 0.00 |
| T4 | 164.22 | 207.00 | 150.00 | 0.00 | 428.40 | 0.00 |
| T5 | 260.55 | 207.00 | 150.00 | 0.00 | 0.00 | 225.00 |

*T1: no fertilizer application (CK); T2: Conventional fertilization (CF; NPK dosage: 714 kg ha^-1^: 357 kg N ha^-1^, 207 kg P ha^-1^, and 150 kg K ha^-1^); T3: 60% CF+12,000 kg ha organic fertilizer (CFO; NPK dosage: 428.4 kg ha^-1^: 214.2 kg N ha^-1^, 124.2 kg P ha^-1^, and 90 kg K ha^-1^); T4: 46% CF+428.4 kg ha humic acid urea (CFO; NPK dosage: 714 kg ha^-1^: 357 kg N ha^-1^, 207 kg P ha^-1^, and 150 kg K ha^-1^); T5: 73% CF+225 kg ha bio-organic fertilizer (CFB; NPK dosage: 617.55 kg ha^-1^: 260.55 kg N ha^-1^, 207 kg P ha^-1^, and 150 kg K ha^-1^)*.
