# Supplementary material for: Chemical fertilizer reduction combined with organic fertilizer affects the soil microbial community and diversity and yield of cotton
Source: Front Microbiol. 2023 Nov 20;14:1295722. doi: 10.3389/fmicb.2023.1295722 (PMC10694218; doi:10.3389/fmicb.2023.1295722)
Supplement: Supplementary file 2 [file Table_2.docx]

Table S2 Soil chemical properties of different treatments

| Sampling time | Treatment | organic matter | total nitrogen | total phosphorus | total potassium | available nitrogen | available phosphorus | available potassium |
| --- | --- | --- | --- | --- | --- | --- | --- | --- |
|  |  | g/kg | g/kg | g/kg | g/kg | mg/kg | mg/kg | mg/kg |
| B | T1 | 10.50+0.65a | 0.61+0.02a | 1.65+0.03b | 16.48+0.55a | 38.71+2.09a | 38.94+1.38b | 118.41+9.01a |
|  | T2 | 10.58+0.63a | 0.59+0.04a | 1.98+0.13a | 14.54+0.78b | 38.67+3.27a | 43.11+3.32a | 130.99+14.31a |
|  | T3 | 10.03+0.55b | 0.51+0.04b | 1.57+0.01b | 11.01+1.10c | 37.76+4.39a | 27.30+0.53b | 131.94+17.62a |
|  | T4 | 11.71+0.34a | 0.66+0.07a | 1.91+0.10a | 8.20+0.73d | 42.68+1.78a | 50.23+1.21a | 114.77+8.58a |
|  | T5 | 10.11+0.23a | 0.60+0.01a | 1.68+0.01b | 6.42+0.35e | 37.03+0.39a | 32.28+3.49b | 124.17+8.82a |
| M | T1 | 11.44+0.83a | 0.54+0.06a | 0.85+0.05a | 16.45+0.35c | 31.48+2.67a | 24.90+6.60a | 101.88+5.97a |
|  | T2 | 11.06+0.52a | 0.54+0.07a | 0.83+0.02a | 16.85+0.08c | 30.43+2.49a | 27.83+2.71a | 107.09+2.04a |
|  | T3 | 10.96+0.36a | 0.64+0.06a | 0.81+0.04a | 17.52+0.05b | 29.52+0.30a | 23.32+5.14a | 105.15+3.62a |
|  | T4 | 10.79+0.18a | 0.60+0.06a | 0.88+0.06a | 17.84+0.15ab | 29.69+1.43a | 30.00+4.42a | 105.27+6.10a |
|  | T5 | 11.22+0.59a | 0.66+0.05a | 0.84+0.04a | 18.00+0.09a | 29.84+0.33a | 31.38+0.70a | 109.84+6.91a |
| H | T1 | 11.74+0.66a | 0.69+0.02ab | 0.85+0.01a | 18.13+0.07a | 30.94+1.62a | 26.14+4.91a | 91.67+4.14a |
|  | T2 | 11.32+0.23a | 0.70+0.08ab | 0.86+0.07a | 18.16+0.13a | 31.52+0.87a | 28.29+5.88a | 95.53+4.68a |
|  | T3 | 10.76+0.50a | 0.65+0.09ab | 0.81+0.05a | 17.85+0.18a | 30.41+0.95a | 28.06+4.90a | 108.46+5.83a |
|  | T4 | 11.11+0.26a | 0.58+0.02b | 0.86+0.05a | 17.50+0.76a | 30.16+1.00a | 24.09+4.54a | 95.13+3.34a |
|  | T5 | 11.18+0.74a | 0.74+0.03a | 0.94+0.09a | 17.89+0.05a | 31.15+1.36a | 36.03+5.45a | 102.45+5.35a |
| T | T1 | 10.93+0.28a | 0.64+0.03a | 0.85+0.03a | 17.67+0.20a | 28.65+0.33b | 22.13+4.11b | 96.39+4.65a |
|  | T2 | 11.02+0.26a | 0.65+0.02a | 0.91+0.08a | 17.50+0.41a | 29.07+1.77ab | 31.75+0.56a | 113.95+4.28a |
|  | T3 | 11.92+0.51a | 0.69+0.09a | 0.88+0.03a | 17.97+0.14a | 32.33+2.66a | 32.35+1.89a | 119.72+5.42a |
|  | T4 | 11.15+0.50a | 0.64+0.03a | 0.87+0.05a | 17.89+0.06a | 29.81+1.32ab | 25.40+5.17ab | 110.83+4.46a |
|  | T5 | 11.42+0.40a | 0.67+0.03a | 0.85+0.03a | 17.73+0.38a | 30.46+1.01ab | 27.49+1.32ab | 115.89+6.55a |

*Values indicate mean ± SE (n = 3). Different superscript letters in the columns represent significant differences among fertilizer treatments according to one-way ANOVA (Duncan’s test, p < 0.05). The abbreviations T1, T2, T3, T4, and T5 are as defined in the footnote to Table S1*. *B: seedling stage; M: bud stage; H: flowering stage; T: boll opening stage.*
