# Supplementary material for: Chemical fertilizer reduction combined with organic fertilizer affects the soil microbial community and diversity and yield of cotton
Source: Front Microbiol. 2023 Nov 20;14:1295722. doi: 10.3389/fmicb.2023.1295722 (PMC10694218; doi:10.3389/fmicb.2023.1295722)
Supplement: Supplementary file 3 [file Table_3.docx]

Table S3 The proportion (%) of bacteria and fungi phylum in co-occurrence network

| Phylum | T1 | T2 | T3 | T4 | T5 |
| --- | --- | --- | --- | --- | --- |
| Bacteria |  |  |  |  |  |
| Proteobacteria | 32.95 | 32.41 | 31.98 | 32.28 | 34.1 |
| Actinobacteriota | 18.77 | 17.00 | 19.03 | 17.72 | 17.24 |
| Chloroflexi | 8.81 | 9.09 | 9.31 | 9.45 | 8.81 |
| Bacteroidota | 8.05 | 6.32 | 6.88 | 7.48 | 7.28 |
| Acidobacteriota | 6.13 | 5.93 | 6.48 | 6.30 | 6.51 |
| Firmicutes | 5.36 | 5.14 | 6.07 | 4.72 | 6.13 |
| Myxococcota | 3.83 | 4.35 | 4.05 | 4.33 | 4.21 |
| Gemmatimonadota | 2.68 | 3.56 | 2.83 | 3.54 | 2.68 |
| Patescibacteria | 2.30 | 2.77 | 2.83 | 2.76 | 1.92 |
| P1anctomycetota | 1.92 | 2.37 | 2.43 | 2.36 | 1.92 |
| Bdellovibrionota | 1.53 | 2.37 | 1.62 | 1.57 | 1.92 |
| Cyanobacteria | 1.53 | 1.58 | 1.21 | 1.57 | 1.53 |
| Fungi |  |  |  |  |  |
| Ascomycota | 80.95 | 78.82 | 77.01 | 73.42 | 77.38 |
| Basidiomycota | 8.33 | 9.41 | 14.94 | 15.19 | 14.29 |
| Chytridiomycota | 2.38 | 2.35 | 2.30 | 3.80 | 2.38 |
| Mortierellomycota | 2.38 | 2.35 | 2.30 | 2.53 | 1.19 |
| G1omeromycota | 2.38 | 2.35 | 2.30 | 1.27 | 1.19 |
| Calcarisporiellomycota | 1.19 | 1.18 | 0.00 | 1.27 | 1.19 |
| unclassified_k_Fungi | 1.19 | 1.18 | 1.15 | 1.27 | 1.19 |
| Rozellomycota | 1.19 | 1.18 | 0.00 | 1.27 | 1.19 |

*The abbreviations T1, T2, T3, T4, and T5 are as defined in the footnote to Table S1*.
