# Supplementary figures and images for: Chemical fertilizer reduction combined with organic fertilizer affects the soil microbial community and diversity and yield of cotton
Source: Front Microbiol. 2023 Nov 20;14:1295722. doi: 10.3389/fmicb.2023.1295722 (PMC10694218; doi:10.3389/fmicb.2023.1295722)

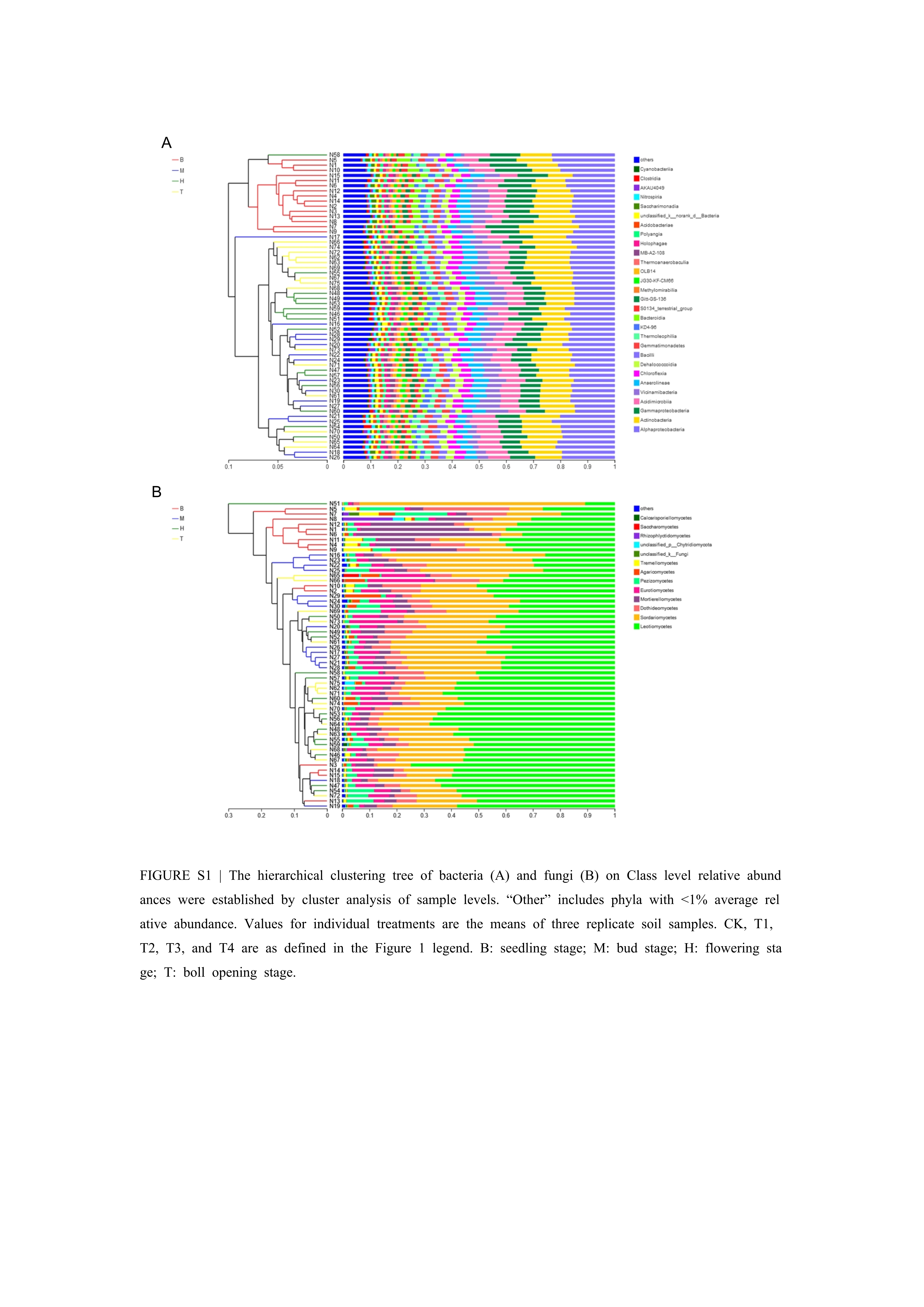

Supplement: Supplementary file 4 [file Image_1.jpg]

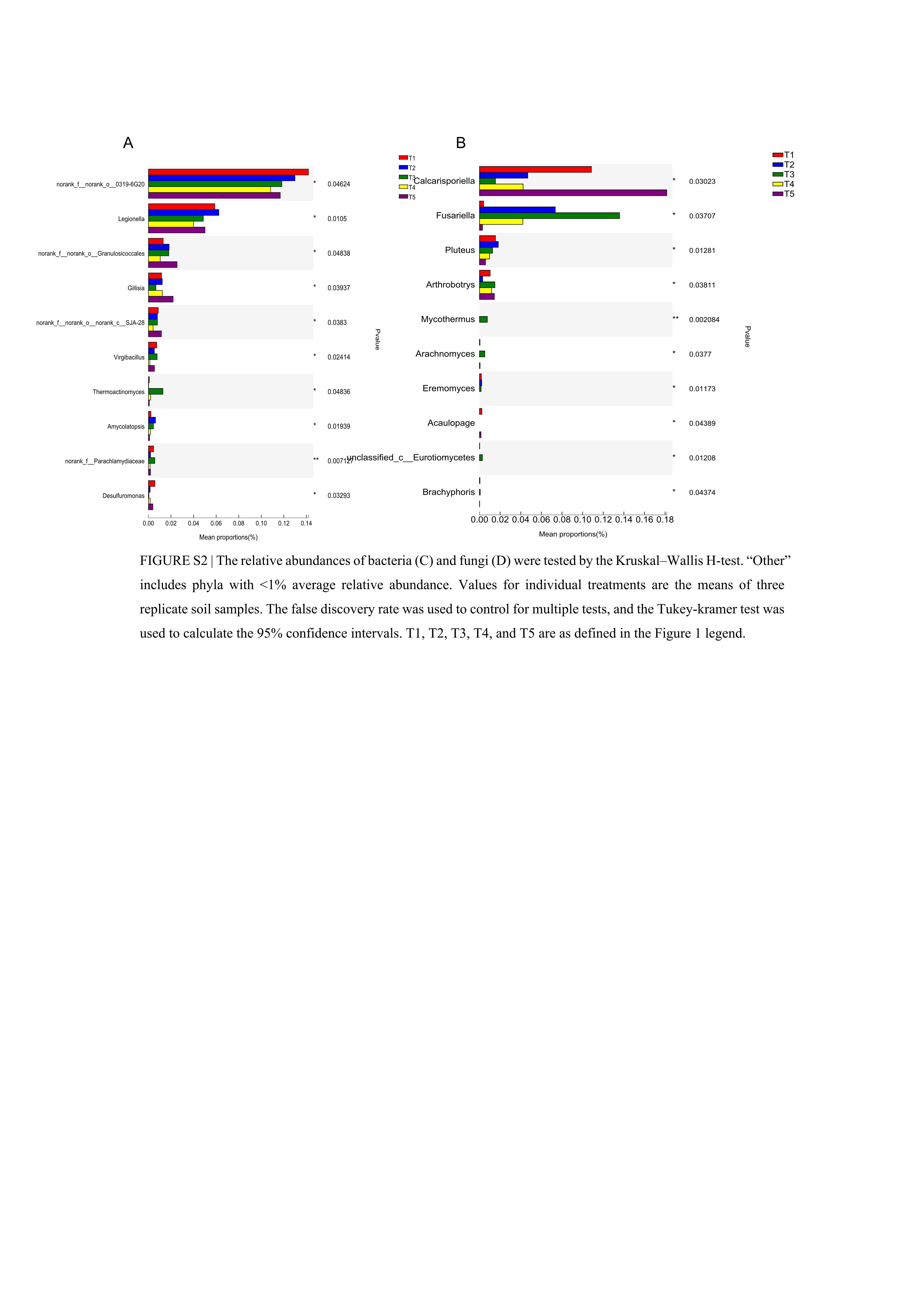

Supplement: Supplementary file 5 [file Image_2.jpg]

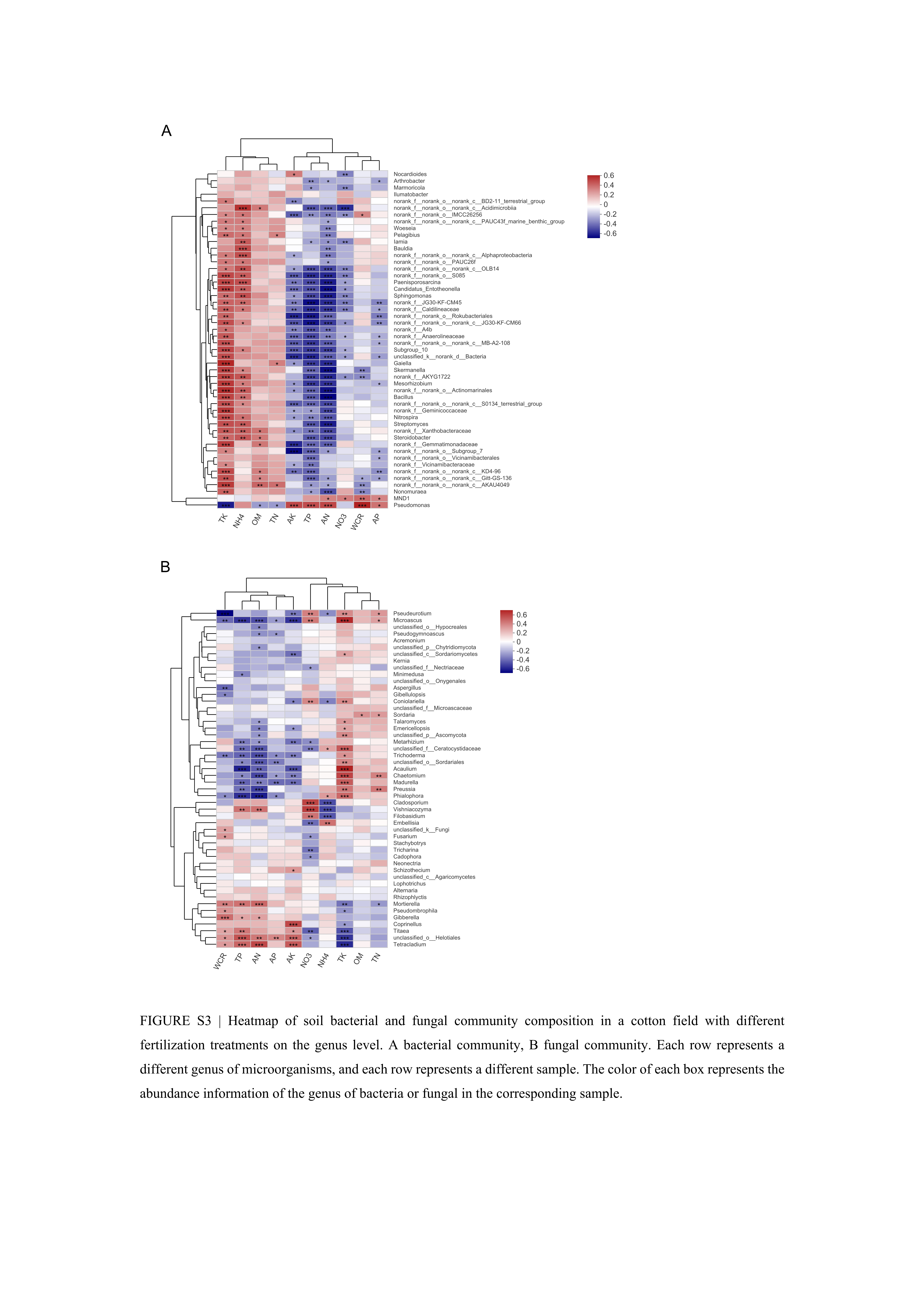

Supplement: Supplementary file 6 [file Image_3.jpg]
